# Improving diagnostic accuracy using a clinical diagnostic support system for medical students during history-taking: a randomized clinical trial

**DOI:** 10.1186/s12909-023-04370-6

**Published:** 2023-05-25

**Authors:** Yasutaka Yanagita, Kiyoshi Shikino, Kosuke Ishizuka, Shun Uchida, Yu Li, Daiki Yokokawa, Tomoko Tsukamoto, Kazutaka Noda, Takanori Uehara, Masatomi Ikusaka

**Affiliations:** Department of General Medicine, Chiba University Hospital, 1-8-1, Inohana, Chuo-Ku, Chiba-City, Chiba Pref Japan

**Keywords:** CDSS, Clinical reasoning, Diagnostic accuracy, Diagnostic error, Google

## Abstract

**Background:**

A clinical diagnostic support system (CDSS) can support medical students and physicians in providing evidence-based care. In this study, we investigate diagnostic accuracy based on the history of present illness between groups of medical students using a CDSS, Google, and neither (control). Further, the degree of diagnostic accuracy of medical students using a CDSS is compared with that of residents using neither a CDSS nor Google.

**Methods:**

This study is a randomized educational trial. The participants comprised 64 medical students and 13 residents who rotated in the Department of General Medicine at Chiba University Hospital from May to December 2020. The medical students were randomly divided into the CDSS group (*n* = 22), Google group (*n* = 22), and control group (*n* = 20). Participants were asked to provide the three most likely diagnoses for 20 cases, mainly a history of a present illness (10 common and 10 emergent diseases). Each correct diagnosis was awarded 1 point (maximum 20 points). The mean scores of the three medical student groups were compared using a one-way analysis of variance. Furthermore, the mean scores of the CDSS, Google, and residents’ (without CDSS or Google) groups were compared.

**Results:**

The mean scores of the CDSS (12.0 ± 1.3) and Google (11.9 ± 1.1) groups were significantly higher than those of the control group (9.5 ± 1.7; *p* = 0.02 and *p* = 0.03, respectively). The residents’ group’s mean score (14.7 ± 1.4) was higher than the mean scores of the CDSS and Google groups (*p* = 0.01). Regarding common disease cases, the mean scores were 7.4 ± 0.7, 7.1 ± 0.7, and 8.2 ± 0.7 for the CDSS, Google, and residents’ groups, respectively. There were no significant differences in mean scores (*p* = 0.1).

**Conclusions:**

Medical students who used the CDSS and Google were able to list differential diagnoses more accurately than those using neither. Furthermore, they could make the same level of differential diagnoses as residents in the context of common diseases.

**Trial registration:**

This study was retrospectively registered with the University Hospital Medical Information Network Clinical Trials Registry on 24/12/2020 (unique trial number: UMIN000042831).

**Supplementary Information:**

The online version contains supplementary material available at 10.1186/s12909-023-04370-6.

## Background

Diagnostic error is “the failure to (a) establish an accurate and timely explanation of the patient’s health problem(s) or (b) communicate that explanation to the patient” [1]. Medical error is estimated as the third leading cause of death in the U.S. after cardiac disease and malignant tumors [2]. Diagnostic errors account for 21% of medical malpractice lawsuits [3]. In Japan, diagnostic errors are the leading cause (54%) of medical malpractice cases [4]. Avoiding diagnostic errors is critical for improving medical care quality.

Most diagnostic errors likely occur during information collection and integration [5]. Japanese residents are prone to making diagnostic errors during history-taking, physical examination, and assessment [6]. Furthermore, 32% of diagnostic errors occur when integrating information from medical history, physical examination, and assessment [7]. In clinical diagnosis, history-taking contributes to diagnosis in about 80% of cases [8], while recalling an appropriate differential diagnosis at the history-taking stage helps avoid diagnostic errors [9] and influences the selection and interpretation of physical examinations and tests based on diseases recalled during history-taking [10].

A clinical decision support system (CDSS) provides important support to recall diseases in medical history for medical students unable to appropriately recall differential diagnoses and experienced doctors experiencing difficulties in making diagnoses [11, 12]. A CDSS both assists novice doctors in recalling diseases and may prevent diagnostic errors due to experienced doctors’ biases [11]. CDSS use has attracted the attention of primary care practitioners [13]. While CDSS use has been known to increase the rate of correct diagnosis among family medicine residents [14], there is also skepticism toward its usefulness for medical students when diagnosing rheumatic diseases [15].

In addition, few studies have examined the use of a CDSS at any point in the diagnostic process. One study compared the diagnostic accuracy of using a CDSS at the point of presentation of a year-old and a chief complaint with that of using a CDSS at the point when all the tests (interview, physical examination, blood tests, imaging tests) had been concluded. The results revealed that using a CDSS when all the information is available is more useful in listing differential diagnoses [16]. However, limited research has examined the accuracy of diagnosis using a CDSS at the history-taking stage. In recent years, artificial intelligence (AI)-driven support systems have also been developed, and reports of high correct response rates for the National Medical Examination in the United States have emerged [17]. However, their output needs to be more reliable and requires the user’s knowledge for accurate evaluation, hindering their practical application. With further improvements in accuracy in the future, AI might compete with CDSS as a diagnostic support system. Google, a web service, has also been studied for its usefulness in supporting diagnoses [18]. To avoid diagnostic errors due to inability to recall a disease, we employed a CDSS and Google.

In this study, we aimed to investigate the diagnostic accuracy of a CDSS, especially in the history-taking phase, for which we divided medical students into three groups: one group using the CDSS, one group using Google, and a control group (using neither the CDSS nor Google). We also compared the CDSS and Google groups and a residents’ group (using neither the CDSS nor Google) to verify the usefulness of the CDSS and Google. Medical students’ diagnostic accuracy was predicted to increase by using the CDSS and Google; these systems’ usefulness was anticipated to be better than that of the residents.

## Methods

### Participants

The participants comprised 64 medical students and 14 residents. Medical students were fifth-year students who participated in a clinical clerkship at the Department of General Medicine, Chiba University Hospital, from May to December 2020. They were rotated in groups, each comprising about 10 students. Residents were in their first and second year and rotated through the same department during the same period. All students and residents were guaranteed that their evaluation would not be affected by their participation in the study.

### Design

**Fig. 1:**
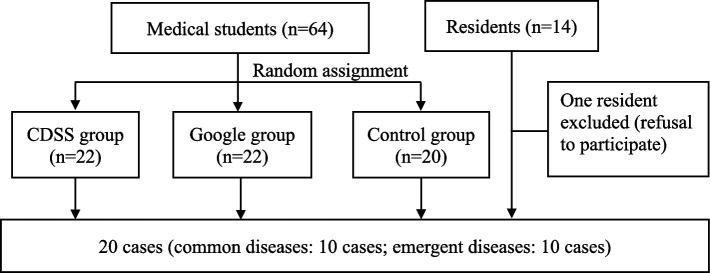
Research flow

The medical students were assigned to one of three groups—the CDSS group, Google group, and control group—by simple randomization using Microsoft Excel 2019 in units of one clinical practice group (Fig. 1). Assignment was unblinded to participants and faculty. Participants answered case questions online. The three medical student groups were compared. Further, we compared the residents’ group with the CDSS and Google groups. This study was a randomized educational trial conducted based on the CONSORT 2010 statement [19].

### Experimental materials

Focus group discussions were held with two supervisors (YY and KS) from the Department of General Medicine for case question development. The questions were based on diseases specified by the Ministry of Health, Labour and Welfare as the objectives of clinical training [20] and the National Medical Examination [21]. Twenty case questions were prepared, with 10 each on common and emergent diseases in the general practice field. Seven physicians in the Department of General Medicine, each with 3–7 years of medical experience, answered the questions correctly at least 80% of the time. The difficulty level of each case was set as easy or difficult (Supplement 1). The case questions mainly comprised age, gender, chief complaint, and medical history (Supplement 2).

### Procedure

Before answering the case questions, the CDSS group was informed that they would be using Current Decision Support^Ⓡ^ (Precision Co., Tokyo, Japan), and their access to the web service was confirmed. This CDSS is used in our hospital and is freely accessible to all medical professionals, including medical students. It is searchable in Japanese and displays differential diagnoses and related symptoms when medical terms (e.g., multiple symptoms) are entered as keywords. The teacher demonstrated to each group how to perform searches and use the CDSS by using the same example.

To standardize the participants’ skills in using Google search, and not Google Scholar, the Google group was also given a demonstration before answering the case questions. The participants were asked to respond based on the information provided by searching for symptom keywords in the case questions. Two teachers participated in this study; one conducted the demonstration before the case questions were answered, and they supervised participants as they answered the case questions. Residents answered the questions without using the CDSS or Google.

### Data collection

Participants individually joined online from a remote location and remained connected with the faculty member while answering the questions. Microsoft Forms (Microsoft Corp., Redmond, DC, USA), a web service, was used to present the case questions. The URL of each case was presented, and participants accessed and answered the case questions using their own devices. Participants were asked to list the top three diseases in order of likelihood. We measured the time required to answer all case questions. One and 0 points were given for each correct and incorrect answer, respectively (maximum score: 20 points). When evaluating the differential diagnoses placed in the first three positions, 1 point was awarded for each correct answer among the three listed diseases. One teacher determined the correct answer. An answer was considered correct if a participant’s response was an exact match to the right name of the disease. Each group’s mean score was compared. As the primary outcome, we compared the mean score of the first position and that of the first three positions in the differentiation of diseases and the response time among the three medical student groups. As the secondary outcome, we evaluated the mean scores of the case questions regarding common diseases, emergent diseases, and difficulty levels by comparing the CDSS, Google, and residents’ groups in the same way.

### Statistical analysis

All statistical analyses were performed using SPSS Statistics for Windows 26.0 (IBM Corp., Armonk, NY, USA). Statistical significance was set to less than 5%. The normality of the data for each group was evaluated with regard to the mean scores of all case questions answered and the time required for answering. If the data were normally distributed, a one-way analysis of variance (ANOVA) was used to compare the CDSS, Google, and control groups. If the data were non-normally distributed, the Kruskal–Wallis test was used. For between-group evaluation, Tukey’s honestly significant difference test was used for the one-way ANOVA, and the Mann–Whitney U-test was used for the Kruskal–Wallis test; *p*-values were modified by Bonferroni correction. We estimated that 42 samples each were necessary for the one-way ANOVA and nonparametric analysis, with a two-sided significance level of 5%, power of 80%, and an effect size of 0.5.

### Ethics approval

The Ethics Review Committee of the Chiba University Graduate School of Medicine approved this study. The researchers verbally obtained the participants’ informed and voluntary consent. Participants were also informed that the data obtained would not be used for university grading and agreed not to share the case questions with other participants.

### Trial registration

This study was registered with the University Hospital Medical Information Network Clinical Trials Registry on 24/12/2020 (unique trial number: UMIN000042831).

## Results

In total, 22, 22, and 20 students were assigned to the CDSS, Google, and control groups, respectively. One resident did not consent to participate; thus, 13 residents were included. The median age of the three student groups was 23.6–24.1 years. The residents’ median age was 26.1 years. By gender, there were 45 men overall (70%), with 17 (77%), 11 (50%), 17 (85%), and eight (62%) men in the CDSS, Google, control, and residents’ groups, respectively (Table 1).

**Table 1:** Participants’ characteristics

	**Control group (*n* = 20)**	**CDSS group (*n* = 22)**	**Google group (*n* = 22)**	**Residents’ group (*n* = 13)**
Age, median (range)	23.9 (22–32)	24.1 (22–34)	23.6 (21–30)	26.1 (25–36)
Gender/men, n (%)	17 (77)	11 (50)	17 (85)	8 (62)

Comparing the three medical student groups, the scores for first position in each group were normally distributed, with a mean of 12.0 ± 1.3, 11.9 ± 1.1, and 9.5 ± 1.7 for the CDSS, Google, and control groups, respectively (Table 2). There was a significant difference between the three medical student groups (*p* = 0.01). Post-hoc comparisons showed that the mean scores of the CDSS and Google groups were significantly higher than those of the control group (*p* = 0.02 and *p* = 0.03, respectively) (Table 3). The difference between the CDSS group and Google group was not statistically significant (*p* = 1.0).

**Table 2:** Mean scores of the three groups of medical students

**Disease differentiation**	**Groups**	**n**	**Mean**	**SD**		**Sum of squares**	**df**	**Mean squares**	**F**	***P* value**
First position	Control	20	9.5	1.7	Between Groups	84.7	2	42.4	4.7	0.01
	CDSS	22	12.0	1.3	Within Groups	549.7	61	9.0		
	Google	22	11.9	1.1						
First three positions	Control	20	10.6	1.6	Between Groups	206.9	2	103.5	15.5	< 0.001
	CDSS	22	14.6	1.0	Within Groups	407.6	61	6.7		
	Google	22	14.4	0.9						

Statistical method: One-way analysis of variance

**Table 3:** Comparison of mean scores among the three medical student groups

Disease differentiation	Compared groups	Mean difference	SE	*P* value	Lower bound	Upper bound
First position	Control vs CDSS	-2.5	0.9	0.02	-4.7	-0.3
	Control vs Google	-2.5	0.9	0.03	-4.7	-0.2
	CDSS vs Google	0.05	0.9	1.0	-2.1	2.2
First three positions	Control vs CDSS	-3.9	0.8	< 0.001	-5.9	-2.0
	Control vs Google	-3.8	0.8	< 0.001	-5.7	-1.9
	CDSS vs Google	0.1	0.8	1.0	-1.7	2.0

Statistical method: Tukey’s honestly significant difference test

The scores of the top three lists in each group were normally distributed, and the means were 14.6 ± 1.0, 14.4 ± 0.9, and 10.6 ± 1.6 for the CDSS, Google, and control groups, respectively. There was a difference between the three medical student groups (*p* < 0.001); the mean scores of the CDSS and Google groups were significantly higher than those of the control group (*p* < 0.001 for each), and the difference between the CDSS and Google groups was not statistically significant (*p* = 1.0).

Upon comparing the CDSS, Google, and residents’ groups, there was a significant difference in the first and first three positions in each group (*p* = 0.006, *p* = 0.01). In the between-group comparison, the mean scores for first position in the differential diagnosis were higher in the residents’ group than in the CDSS and Google groups (*p* = 0.01 and *p* = 0.01, respectively).

Regarding the 10 common disease cases, the mean scores for first position in the differential diagnosis were 7.4 ± 0.7, 7.1 ± 0.7, and 8.2 ± 0.7 for the CDSS, Google, and residents’ groups, respectively. There was no significant difference in the mean scores (*p* = 0.1). The mean scores for the first three positions in the differential diagnosis were 8.3 ± 0.5, 8.3 ± 0.5, and 8.6 ± 0.6 for the CDSS, Google, and residents’ groups, respectively. There was no significant difference in the mean scores (*p* = 0.7) (Table 4).

**Table 4:** Comparison of mean scores for common, emergent, easy, and difficult disease cases

	**Differential diagnosis**	**CDSS group** **(*n* = 22)**	**Google group (*n* = 22)**	**Residents’ group** **(*n* = 13)**	***P* value**
Common	First position	7.4 ± 0.7	7.1 ± 0.7	8.2 ± 0.7	0.1^†^
	First three positions	8.3 ± 0.5	8.3 ± 0.5	8.6 ± 0.6	0.7^†^
Emergent	First position	4.7 ± 0.8	4.9 ± 0.8	6.7 ± 1.0	0.004^‡^
	First three positions	6.4 ± 0.7	6.2 ± 0.8	8.1 ± 1.1	0.006^†^
Easy	First position	6.2 ± 0.9	6.6 ± 0.6	7.7 ± 0.9	0.04^†^
	First three positions	7.8 ± 0.7	7.8 ± 0.6	8.5 ± 0.8	0.3^†^
Difficult	First position	5.8 ± 0.7	5.3 ± 0.9	7.0 ± 0.8	0.02^†^
	First three positions	6.8 ± 0.6	6.6 ± 0.7	8.0 ± 0.9	0.02^†^

Statistical method: †Kruskal–Wallis test, ‡One-way analysis of variance

For the 10 emergent cases, the mean scores were significantly higher in the residents’ group than in the CDSS and Google groups (*p* = 0.005 and *p* = 0.01, respectively). The difference between the CDSS and Google groups was not statistically significant (*p* = 0.9) (Table 5).

**Table 5:** Evaluation of 10 emergent cases between the CDSS, Google, and residents’ groups

Differential diagnosis	Compared groups	*P* value
First position	CDSS vs Google	0.9^†^
	CDSS vs Residents	0.005^†^
	Google vs Residents	0.01^†^
First three positions	CDSS vs Google	1.0^‡^
	CDSS vs Residents	0.01^‡^
	Google vs Residents	0.01^‡^

Statistical method: †Tukey’s honestly significant difference test, ‡Dunn–Bonferroni post hoc test

For the mean scores for the first three positions in the differential diagnosis, the mean score for the residents’ group was significantly higher than the scores for the CDSS and Google groups (both *p* = 0.01). The difference between the CDSS and Google groups was not statistically significant (*p* = 1.0).

In the evaluation of easy case questions, the mean scores for the diseases placed in the first three positions in the CDSS, Google, and residents’ groups showed no significant difference (*p* = 0.3) (Table 4). In the difficult case questions, the mean scores for the diseases placed in the first three positions in the CDSS, Google, and residents’ groups showed a significant difference between the CDSS and residents’ groups and between the Google and residents’ groups (*p* = 0.04 and *p* = 0.02, respectively) (Table 6).

**Table 6:** Evaluation of scores by difficulty level between the CDSS, Google, and residents’ groups

Difficulty level	Differential diagnosis	Compared groups	*P* value
Easy	First position	CDSS vs Google	1.0
		CDSS vs Residents	0.03
		Google vs Residents	0.2
Difficult	First position	CDSS vs Google	1.0
		CDSS vs Residents	0.1
		Google vs Residents	0.02
	First three positions	CDSS vs Google	1.0
		CDSS vs Residents	0.04
		Google vs Residents	0.02

Statistical method: Dunn–Bonferroni post hoc test

The total time taken to answer all case questions was 5,253, 5,587, 3,652, and 3,956 s for the CDSS, Google, control, and residents’ groups, respectively. The CDSS and Google groups showed significantly longer answering times than the control group (*p* < 0.001, *p* < 0.001, respectively). The CDSS and Google groups also had significantly longer answering times than the residents’ group (*p* = 0.2 and *p* = 0.01, respectively). There was no significant difference in answering time between the CDSS and Google groups (*p* = 1.0).

## Discussion

In this study, we showed that medical students tended to make diagnoses more accurately using the CDSS and Google for typical disease history, which residents should learn. For common diseases, there was no significant difference in the mean scores of correct answers between medical students, who used the CDSS and Google, and residents, indicating the usefulness of the CDSS and Google. For emergent diseases, the mean scores of medical students who used the CDSS and Google were not as high as those of residents.

For easy cases, the scores of medical students using the CDSS or Google were equivalent to those of residents. For difficult cases, medical students did not reach the average score of residents even when using the CDSS and Google and when within the first three position differentials. The residents’ mean scores were almost equal for common and emergent diseases; this is possibly because they have a better understanding of disease concepts and clinical processes through clinical practice. Additionally, residents’ lower mean scores for emergent diseases may indicate that the case questions were more complicated than those for common diseases. Furthermore, as medical students mainly rotate through wards and outpatient departments, they have few opportunities to experience emergent disease care.

The quality of information in the CDSS is generally assured as the medical information provided is reflective of relevant guidelines and expert opinions [22]. The displayed information is organized in order of common diseases, emergent diseases, and frequency, making it easy to understand. In addition, the information the CDSS presents is concise and described in medical terms, aiding medical professionals’ understanding. On the contrary, while Google is free of charge, its algorithm displays overlapping medical information from a mixture of sources (e.g., hospitals, promotion). Therefore, the information is less reliable [23], and there is no guarantee of accuracy. The similarity between the CDSS and Google is that medical students using both systems were not as accurate as residents in answering the difficult case questions. The reason may relate to the students’ ability to set appropriate search keywords and semantic qualifiers. Moreover, skills to evaluate and select the usefulness of the information presented as search results may have been lacking because they were novice users.

No significant difference was found between the CDSS and Google groups regarding answering time. Searching with the CDSS is time-consuming and requires training to become familiar with its use. Accordingly, higher frequency of use, continued use, and increased proficiency in the CDSS are expected to help avoid diagnostic errors and improve diagnostic accuracy in daily practice. Accuracy of diagnosis decreases when time is limited [24]. In this study, as the CDSS was used without time limitation, diagnostic accuracy was not affected by time.

One study evaluated the diagnostic accuracy of the CDSS at the time the patient presents with the original complaint and at the time all test results are available, including the interview, physical examination, blood tests, and imaging studies [16]. The appropriateness of the use of the CDSS after a certain amount of information has been gathered was consistent with the present study in that it identified the appropriate differential disease. The difference is that our study used only the medical history, excluding physical examination and laboratory findings information, which is different from the data entered into the CDSS. The disease recalled from the medical history information will affect the subsequent physical examination and laboratory tests to be undertaken. Previous reports have acknowledged that diagnostic errors are likely to occur at this point [6].

There are two issues that remain to be discussed. First, the accuracy of the CDSS must be further improved. Currently, the CDSS is reported to be sufficiently accurate in the family medicine field [14], but it may be less useful for certain diseases [15]. Patients may be disadvantaged if the accuracy of the CDSS is not ensured [25]. As shown in previous studies, the accuracy of Google is high when using characteristic symptoms as search terms, making it a helpful diagnostic system for common diseases. However, the possibility of accurately searching for diseases with multiple nonspecific symptoms is reduced [25]. In this study, the rate of correct answers was higher in the Google group than in the control group, and the Google and CDSS groups had no significant differences.

Second, the CDSS requires a certain proficiency level, especially in the search method and appropriate selection of keywords, whereas the participants were accustomed to using Google regularly. Another advantage of Google is that any keywords can be used, without any constraints; they do not have to be medical terms. Meanwhile, with the CDSS, users’ basic medical knowledge would help them select appropriate medical terms and evaluate the information appropriately. As the next research step, we would like to assess the effect of CDSS use on the performance of more experienced clinicians. Clarifying the usefulness of a CDSS could guide learning on how to use medical information systems in medical education. Moreover, ChatGPT (OpenAI, San Francisco), an AI-driven system, was released at the end of 2022 and has reportedly achieved a high percentage of correct answers in the US National Medical Examination [17]. We consider that this newly introduced AI-driven system must receive attention as CDSS competitors, and it is easy to predict that with improved information literacy, including the selection of input content and evaluation of output information, the AI-driven system might dominate in the field of CDSS in the future.

### Limitations

This study has several limitations. First, it was conducted at a single institution, and the results might not be generalizable as the proficiency levels of students and residents were not assessed. Second, as the allocation to the three groups could not be blinded for participants and faculty members, subjective bias may have affected the results. Third, the case questions were set with reference to diseases as defined in the attainment objectives of clinical training provided by the Ministry of Health, Labour and Welfare and the National Medical Examination. From the viewpoint of difficulty level, it is possible that the diagnoses could have been made quickly using the CDSS based on specific symptoms. Moreover, we did not verify the usefulness of the CDSS in more complex cases. Fourth, as the study period was from May to December, there may be differences in clinical exposure and training between those who participated in the first and second half of the study. Fifth, as there was no set response time for answering the cases, it is difficult to determine whether the CDSS and Google groups spent more time using the system or pondering each question to explain their longer response times than the control group. Finally, the case questions were described in medical terms so that the symptoms could be easily grasped and retrieved. These questions differed from patients’ complaints in actual clinical practice, which in turn require practitioners/students to convert the patients’ complaints into medical terms that can be searched using the CDSS; this conversion process may lead to errors. It is essential to be able to extract appropriate keywords and verify whether these can be converted into medical terms and retrieved.

## Conclusions

When using the CDSS or Google, medical students can make more accurate differential diagnoses than when not using either. Although the diagnostic accuracy of medical students using the CDSS was not as high as that of residents regarding emergency diseases and complex cases, it was similar to that of residents pertaining to common diseases.

## Supplementary Information

**Additional file 1: Supplement 1.** Case lists classification.

**Additional file 2: Supplement 2.** Case samples.

## Data Availability

The protocol and the case questions used during the current study are available from the corresponding author on reasonable request.

## Abbreviations

AI: Artificial intelligence
ANOVA: Analysis of variance
CDSS: Clinical diagnostic support system
