# Correction to: Improving diagnostic accuracy using a clinical decision support system for medical students during history-taking: a randomized clinical trial

**DOI:** 10.1186/s12909-023-04468-x

**Published:** 2023-06-27

**Authors:** Yasutaka Yanagita, Kiyoshi Shikino, Kosuke Ishizuka, Shun Uchida, Yu Li, Daiki Yokokawa, Tomoko Tsukamoto, Kazutaka Noda, Takanori Uehara, Masatomi Ikusaka

**Affiliations:** Department of General Medicine, Chiba University Hospital, 1-8-1, Inohana, Chuo-Ku, Chiba-City, Chiba Pref Japan

**Correction to: BMC Medical Education (2023) 23:383**

10.1186/s12909-023-04370-6

Following publication of the original article [1], due to a typesetting misunderstanding, it has been changed the word “diagnostic” to “decision” in the title.

The article title is corrected above and the original article has been corrected.
