# Supplementary material for: Improving diagnostic accuracy using a clinical diagnostic support system for medical students during history-taking: a randomized clinical trial
Source: BMC Med Educ. 2023 May 25;23:383. doi: 10.1186/s12909-023-04370-6 (PMC10214648; doi:10.1186/s12909-023-04370-6)
Supplement: Supplementary file 1 — Additional file 1: Supplement 1. Case lists classification. [file 12909_2023_4370_MOESM1_ESM.docx]

Supplement 1) Case lists classification

| No. | Disease | common | emergency | difficult | easy |
| --- | --- | --- | --- | --- | --- |
| 1 | Transient cerebral ischemia |  | ◯ |  | ◯ |
| 2 | Acute pyelonephritis | ◯ |  | ◯ |  |
| 3 | Gastroesophageal reflux disease | ◯ |  | ◯ |  |
| 4 | Panic disorder | ◯ |  | ◯ |  |
| 5 | Lumbar spinal canal stenosis | ◯ |  |  | ◯ |
| 6 | Benign Paroxysmal Positional Vertigo | ◯ |  |  | ◯ |
| 7 | Knee osteoarthritis | ◯ |  | ◯ |  |
| 8 | Parkinson's disease | ◯ |  | ◯ |  |
| 9 | Acute sinusitis | ◯ |  |  | ◯ |
| 10 | Acute lumbago | ◯ |  | ◯ |  |
| 11 | Acute epiglottitis |  | ◯ |  | ◯ |
| 12 | Crowned dens syndrome | ◯ |  |  | ◯ |
| 13 | Subarachnoid hemorrhage |  | ◯ | ◯ |  |
| 14 | Cholecystitis |  | ◯ | ◯ |  |
| 15 | Acute pancreatitis |  | ◯ | ◯ |  |
| 16 | Pulmonary thromboembolism |  | ◯ |  | ◯ |
| 17 | Acute epicarditis |  | ◯ |  | ◯ |
| 18 | Ectopic pregnancy |  | ◯ |  | ◯ |
| 19 | Meningitis |  | ◯ | ◯ |  |
| 20 | Acute angle closure glaucoma |  | ◯ |  | ◯ |
