# Supplementary material for: Improving diagnostic accuracy using a clinical diagnostic support system for medical students during history-taking: a randomized clinical trial
Source: BMC Med Educ. 2023 May 25;23:383. doi: 10.1186/s12909-023-04370-6 (PMC10214648; doi:10.1186/s12909-023-04370-6)
Supplement: Supplementary file 2 — Additional file 2: Supplement 2. Case samples. [file 12909_2023_4370_MOESM2_ESM.docx]

Supplement 2) Case samples

Common disease)

Two weeks before the appointment, a 36-year-old man had nasal discharge, a cough, and a fever. Despite taking over-the-counter medications, his symptoms subsided for around three days. He came to the clinic five days before the appointment because he had purulent nasal discharge, nasal discharge that ran down his mouth, and soreness in the area of his right cheek.

No prior medical history.

Emergency disease）

A 3-year-old boy's overall health was okay, but he had a fever and sore throat for three days. He was unable to eat the morning of the visit due to a sore throat, and he subsequently started to cough and slobber without swallowing. His mother brought him to the clinic for an examination.

No prior medical history.
